# Levels and Determinants of Prenatal Breastfeeding Knowledge, Attitude, and Intention Among Pregnant Women: A Cross-Sectional Study in Northwest Ethiopia

**DOI:** 10.3389/fpubh.2022.920355

**Published:** 2022-07-15

**Authors:** Endeshaw Chekol Abebe, Gebrehiwot Ayalew Tiruneh, Getachew Asmare Adela, Teklie Mengie Ayele, Zelalem Tilahun Muche, Awgichew Behaile T/Mariam, Anemut Tilahun Mulu, Tadesse Asmamaw Dejenie

**Affiliations:** ^1^Department of Biomedical Sciences, College of Health Sciences, Debre Tabor University, Debre Tabor, Ethiopia; ^2^Department of Clinical Midwifery, College of Health Sciences>, Debre Tabor University, Debre Tabor, Ethiopia; ^3^Department of Reproductive Health and Nutrition, School of Public Health, Woliata Sodo University, Woliata Sodo, Ethiopia; ^4^Department of Pharmacy, College of Health Sciences, Debre Tabor University, Debre Tabor, Ethiopia; ^5^Department of Medical Biochemistry, College of Medicine and Health Sciences, University of Gondar, Gondar, Ethiopia

**Keywords:** knowledge, attitude, intention, pregnant women, breastfeeding, DTCSH, Ethiopia

## Abstract

**Background:**

Pregnant women are a critical part of the community to assess various determinants of their future breastfeeding practice. This study aimed to assess the levels and determinants of breastfeeding knowledge, attitude, and intention among pregnant women.

**Methods:**

A hospital-based cross-sectional study was conducted among 422 pregnant women from January 18 to February 27, 2022, at Debre Tabor Comprehensive Specialized Hospital (DTCSH) in Northwest Ethiopia. Data were collected *via* face-to-face interviews from participants selected by convenience sampling technique. Data analysis was made using Stata version 16.0. Multiple logistic regression analysis was used to assess the determinants of the knowledge, attitude, and intention of pregnant women, with a *p*-value < 0.05 considered statistically significant.

**Result:**

About 57.8% of participants had adequate breastfeeding knowledge and only 46.9% had a positive attitude. Almost two-thirds (65.4%) of them had good intentions to breastfeed. Pregnant women attaining secondary education (AOR = 2.0; 95% CI: 1.31, 3.19), achieving college or university education (AOR = 3.13; 95% CI: 1.63, 7.41), being multiparous (AOR = 2.11; 95% CI: 1.33, 3.43), having four or more ANC visits (AOR:1.45; 95% CI: 1.21, 4.31), and having prior breastfeeding experience (AOR: 3.53; 95% CI: 2.22, 5.65) were significant predictors of adequate knowledge. Attending college or university education (AOR = 2.71;95% CI: 2.33, 5.13), being multiparous (AOR = 1.56; 95% CI: 1.32, 8.25), and having adequate knowledge (AOR = 2.02; 95% CI: 1.88,7.14) were determinants of a positive breastfeeding attitude. Whereas, advanced age (AOR = 1.44; 95% CI: 1.12, 5.59), adequate knowledge (AOR: 5.21; 95% CI: 1.51,8.04), and positive attitude (AOR = 2.41;95% CI:1.50, 4.27) were independent predictors of good breastfeeding intention.

**Conclusion:**

The breastfeeding knowledge and attitude of pregnant women were generally suboptimal. Their overall breastfeeding intention was also unsatisfactory. This highlights the need to develop culture-specific interventions aimed at improving breastfeeding knowledge, attitudes, and intention to enhance the appropriate breastfeeding practice of their future children.

## Introduction

Appropriate breastfeeding practice involves exclusive breastfeeding (EBF) of infants for the first 6 months of life and continuing breastfeeding up to 2 years or beyond, with the introduction of complementary food at the age of 6 months (1). Appropriate breastfeeding practice is the most effective global public health intervention to ensure optimal health and development in infants (2–4). It decreases the risk of developing illnesses and disease severity during childhood, and has the highest life-saving potential by avoiding half a million infant deaths and 13% of child deaths globally (5–8).

Even though appropriate infant feeding practice is the most cost-effective intervention, its global implementation is still suboptimal (9, 10). Suboptimal infant feeding practices adversely affect the child's health and nutritional status, mainly in the first year of life (11). Breastfeeding in Ethiopia is a common practice by the majority of mothers, but appropriate breastfeeding remains disproportionately low. Nearly 70% of infants in Ethiopia were reported to be suboptimally breastfed and 24% of infant mortality and 58% of child mortality were attributed to poor and inappropriate breastfeeding practice (12, 13). This may be due to various factors affecting optimal breastfeeding traditions.

Appropriate breastfeeding practice is highly influenced by various psychosocial factors, including breastfeeding knowledge, attitude, and intention/decision (14, 15). Women begin motherhood with infant feeding knowledge and attitudes that they have adopted passively throughout their lives as part of their larger social, cultural, and environmental context (16). This concept may also be supplemented during pregnancy by prenatal advice from health professionals. More importantly, proactive personal information-seeking during pregnancy and early infancy may lead to the acquisition of knowledge and attitudes (17). The acquired maternal knowledge and attitudes toward breastfeeding are likely to influence their breastfeeding decisions and ultimately their breastfeeding behavior (18).

Breastfeeding intention is regarded as a primary driver that determines women's breastfeeding behavior. Health behaviors, in general, and breastfeeding practice, in particular, are unlikely to occur without the intention of attaining them (19–24). Based on the Theory of Planned Behavior and Theory of Reasoned Action, the intention is found to be a good predictor of behavior that plays a critical role in determining breastfeeding behavior (20, 25). Breastfeeding decisions of the future mothers are usually made before conception per their socio-cultural context before the birth of the baby, during pregnancy, or even before (26). The stronger the intention to breastfeed during the prenatal period, the more likely the mother will exclusively breastfeed after the baby is born (27). However, intention by itself is many steps distant from implementation since various individual-level variables are known to affect breastfeeding decisions, including women's sociodemographic factor and their current breastfeeding knowledge and attitude (28, 29). Thus, breastfeeding intention and its antecedents are modifiable factors that can be used as a point of programmatic intervention to enhance breastfeeding (30–32).

Several studies on breastfeeding knowledge, attitude, and intention have been undertaken among pregnant women globally (19, 33–38). But there is a paucity of data in Ethiopia that shows the levels of breastfeeding knowledge, attitude, and intention among pregnant women. Hence, this study aimed to assess breastfeeding knowledge, attitude, intention, and determinants among pregnant women. Gaining a deeper insight on levels and specific determinants of breastfeeding knowledge, attitude, and intention during pregnancy is crucial for policymakers to design area-appropriate programmatic interventions aimed to foster the mothers' preparation for breastfeeding.

## Methods and Materials

### Study Design, Period, and Setting

A hospital-based cross-sectional study was done from January 18 to February 27, 2022, at the Debre Tabor Comprehensive Specialized Hospital (DTCSH) in Debre Tabor Town, Northwest Ethiopia. DTCSH is currently providing comprehensive healthcare services for about 2.3 million people. It comprises outpatient, inpatient, and emergency departments, delivering antenatal care (ANC), delivery, laboratory, ART, psychiatry, pharmacy, radiology, and other services. This study was conducted particularly in the maternal and child health (MCH) clinic of DTCSH among pregnant women who came for ANC service.

### Population

All pregnant women who had attended the MCH clinic of DTCSH for ANC service were considered as the source population, while all pregnant women who visited the MCH clinic of DTCSH and who were available during the study period were taken as the study population.

### Inclusion and Exclusion Criteria

All volunteer pregnant women (aged 18 years or above) who came for ANC visits at DTCSH were included in this study. However, women who had severe medical or psychiatric illnesses were excluded from the study. Besides, pregnant women aged <18 years were excluded from the study, since their educational level is lower and their knowledge, attitude, and decision-making ability about breastfeeding are most likely different from that of older women.

### Study Variables

Breastfeeding knowledge, attitude, and intention of participants were taken as dependent variables. But sociodemographic characteristics (age, marital status, religion, ethnicity, education, occupation, and residence) and obstetric history (gestational age, gravidity, parity, medical illness, number of ANC visits, breastfeeding experience, place of delivery, and mode of delivery of their last-child) were considered as independent variables in all regression analyses. Whereas, breastfeeding knowledge was taken as an independent variable during the analysis of attitude and intention as outcome variables. The attitude was considered as a predictor variable to check its association with breastfeeding intention.

### Sample Size Determination and Sampling Procedures

The sample size was calculated using the single population proportion formula:


$$n=\frac{{\left(Z_{1-\alpha /2}\right)}^{2}P(1-P)}{d^{2}}$$

Where *n* is the sample size; *Z*_1−α/2_ is the standard normal variable, at 95% confidence level = 1.96; *P* is the estimated proportion of mothers with adequate breastfeeding knowledge (*P* = 52.4%) from a prior study (36); and d is the margin of error = 0.05. The calculated sample size was 422 after adding a 10% non-response rate. A convenience sampling technique was used to recruit participants during data collection.

### Data Collection Tools and Procedure

Data were collected using a pretested questionnaire prepared in the local Amharic language through face-to-face interviews by three trained nurses normally working at the MCH clinic of DTCSH. The questionnaire was designed to collect data on sociodemographic characteristics, obstetric history, and prior breastfeeding experience of respondents. Besides, the questionnaire involved the validated Amharic version of the Breastfeeding Knowledge Questionnaire (BFKQ-A) to measure their knowledge, Iowa Infant Feeding Attitude Scale **(**IIFAS-A) to assess attitude, and locally adapted questions for breastfeeding intention (39).

The BFKQ-A consists of 10 multiple-choice questions and 10 true-false questions, with scores ranging from 0 to 20 and higher scores indicating better knowledge about breastfeeding. The BFKQ-A measure has been shown to have adequate reliability in the Ethiopian context, with Cronbach's α of 0.81 and 0.79 in our evaluation and previous Ethiopian study, respectively (39). Participants who responded to the “correct” answers to any of the 20 questions were given a score of 1 and “wrong” responses to any of these questions were given a score of 0. The total BFKQ-A score of all respondents was categorized as adequate knowledge, if they scored the median value (correctly answered 10 questions) of the total BFKQ-A scale or above, otherwise considered as having inadequate knowledge of breastfeeding (36).

On the other hand, the validated Amharic version IIFAS-A consists of 17 items with a five-point Likert scale, ranging between 1 (strongly disagree) and 5 (strongly agree). The scale was found to be a valid and reliable measure of an individual's attitude toward breastfeeding in the Ethiopian context, with acceptable internal consistency in this study (Cronbach's α = 0.80) and a prior study in Ethiopia (Cronbach's α = 0.72) (39). The IIFAS score ranges from 17 to 85 and the attitudes of pregnant women were classified as a positive attitude (if the IIFAS score **≥**70) and a negative attitude (if the IIFAS score <70) toward breastfeeding (14).

The breastfeeding intention of participants was assessed using a questionnaire constructed by reviewing other published literature (28, 40), and consisted of eight-point intention scale questions, with scores ranging from 0 to 8. A score of 1 point was given for each correct response and 0 for any incorrect or “undecided” responses to each question. The overall score was categorized into good breastfeeding intention, when participants scored ≥ midpoint of the scale (median intention scale = 4), and poor breastfeeding intention, when participants scored below the midpoint of the scale. Cronbach's alpha for this set of questions was 0.83, indicating an acceptable internal consistency.

### Data Processing and Analysis

The collected data were first checked for completeness and internal consistency and then cleaned data were entered into Epi Info version 7.2.4.0 and exported into Stata version 16.0 software for statistical analysis. Summary statistics were reported using frequency and percentage for categorical variables and using mean and standard deviation (SD) to describe continuous variables. A logistic regression model was used to identify determinants of breastfeeding knowledge, attitude, and intentions. Those variables with a *p*-value of 0.2 in simple regression were entered into multiple regression models. Using multiple logistic regression, the results were expressed as adjusted odds ratio (AOR) with 95% confidence intervals (CI) and *p*-values. *P*-values <0.05 were considered statistically significant.

### Data Quality Management

Data were collected using a structured questionnaire prepared in English and translated into Amharic and then retranslated back into English to check its consistency. Questionnaires were checked and made comments for content validity by an expert panel. In addition, to ascertain the understanding, validity, and reliability of the questionnaire and to examine practical issues in selecting participants, a pilot study was carried out before the actual data collection period among 25 pregnant women in Addis Zemen Primary Hospital. Before the initiation of data collection, extensive training was given to data collectors and supervisors to enable them to have an understanding of the objectives of the study and each of the questionnaires. All the data collection was done by three trained nurses under the supervision of two trained supervisors during the data collection period. Questionnaires were checked daily for completeness, and daily feedback on data collection activities was given by supervisors.

### Ethical Approval and Consent to Participate

The study was conducted after getting ethical approval from the Ethical Review Committee of Debre Tabor University and also a written collaboration letter was obtained from the DTCSH administrative office. Written informed consent was taken from each selected participant after a brief explanation of the study objective. Participants were also informed that participation is voluntary that they can withdraw from the study at any time and that their decision to continue or not in the study will not influence their provision of healthcare services. Confidentiality of information as well as anonymity of the data collection procedure were kept. Appropriate infection prevention measures and principles related to COVID-19 were also considered during data collection.

## Results

### Sociodemographic Characteristics

A total of 422 participants have completed the questionnaire and were included in this study. The mean (±SD) age of the respondents was 30.1 ± 5.02 years and the age ranged from 18 to 51 years. Most of the participants were Orthodox Christian 408 (96.7%), married 412 (97.6%), and Amhara ethnicity 416 (98.6%). The greatest proportion of respondents had achieved primary education 167 (39.6%) and a sizable portion were housewives 187 (44.3%) and urban dwellers 273 (64.3%) (Table 1).

**Table 1:** Sociodemographic characteristics of pregnant women attending the MCH clinic of DTCSH in Debre Tabor, Northwest Ethiopia, 2022.

**Variable**	**Category**	**Frequency (*n =* 422)**	**Percent (%)**
Age (years)	<25	47	11.1
	25–34	157	37.2
	35–44	180	42.7
	**≥**45	38	9.0
Religion	Orthodox	408	96.7
	Muslim	8	1.9
	Protestant	6	1.4
Marital status	Married	412	97.6
	Single	3	0.7
	Divorced	6	1.4
	Widowed	1	0.2
Educational status	No formal education	98	23.2
	Primary education	167	39.6
	Secondary education	135	32.0
	College/University	22	5.2
Occupation	Housewife	187	44.3
	Merchant	148	35.1
	Government employee	69	16.4
	Private employee	12	2.8
	Student	4	0.9
	Daily laborer	2	0.5
Ethnicity	Amhara	416	98.6
	Tigray	3	0.7
	Oromo	2	0.5
	Other	1	0.2
Residence	Urban	313	74.2
	Rural	109	28.8

### Obstetric and Breastfeeding History

A total of 371 (87.9%) women were multigravida who had a history of two or above pregnancies, including the current one. About 57 (13.5%) participants were nulliparous who never had a live birth, but the majority 365 (86.5%) were primiparous who had a prior history of one live birth and multiparous having two or above live births. Almost all 415 (98.3%) respondents had planned current pregnancy. Nearly half 208 (49.3%) of them were in the third trimester (≥28 weeks) of the current pregnancy. Above half 245 (58.1%) of women were having <4 ANC visits at the time of the interview. Around 94.1% of them replied that they did not experience any medical illness during their current pregnancy. Of those women who had a prior history of delivery, most of them gave birth to their last child vaginally (90.1%) in health institutions (86.6%). About 86.5% had prior breastfeeding experience (Table 2).

**Table 2:** Obstetric and breast-feeding history of pregnant women attending the MCH clinic of DTCSH in Debre Tabor, Northwest Ethiopia, 2022.

**Variables**	**Category**	**Frequency (*n =* 422)**	**Percent (%)**
Number of pregnancies (gravidity)	Primigravida	51	12.1
	Multigravida	371	87.9
Number of live births (parity)	Nulliparous	57	13.5
	Primiparous	79	18.7
	Multiparous	286	67.8
The current pregnancy is planned	Yes	415	98.3
	No	7	1.7
Gestational age for current pregnancy	1st trimester (<13 week)	50	11.8
	2ndtrimester (13–27 week)	164	38.9
	3rd trimester (≥28 weeks)	208	49.3
Number of ANC visits	<4	245	58.1
	≥4	177	41.9
Medical illness during the current pregnancy	Yes	25	5.9
	No	397	94.1
Place of delivery of their last child (*n =* 365)	Home	49	13.4
	Health institution	316	86.6
Mode of delivery of their last child (*n =* 365)	Vaginal delivery	329	90.1
	Cesarean delivery	36	9.9
Previous breastfeeding experience	Yes	365	86.5
	No	57	13.5

### Knowledge About Breastfeeding

**Figure 1:**
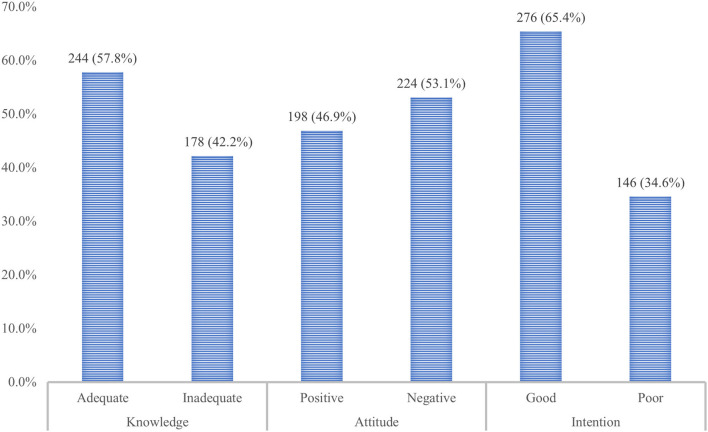
The overall breastfeeding knowledge, attitude, and intention of pregnant women attending the MCH clinic of DTCSH in Debre Tabor, Northwest Ethiopia, 2022.

**Figure 2:**
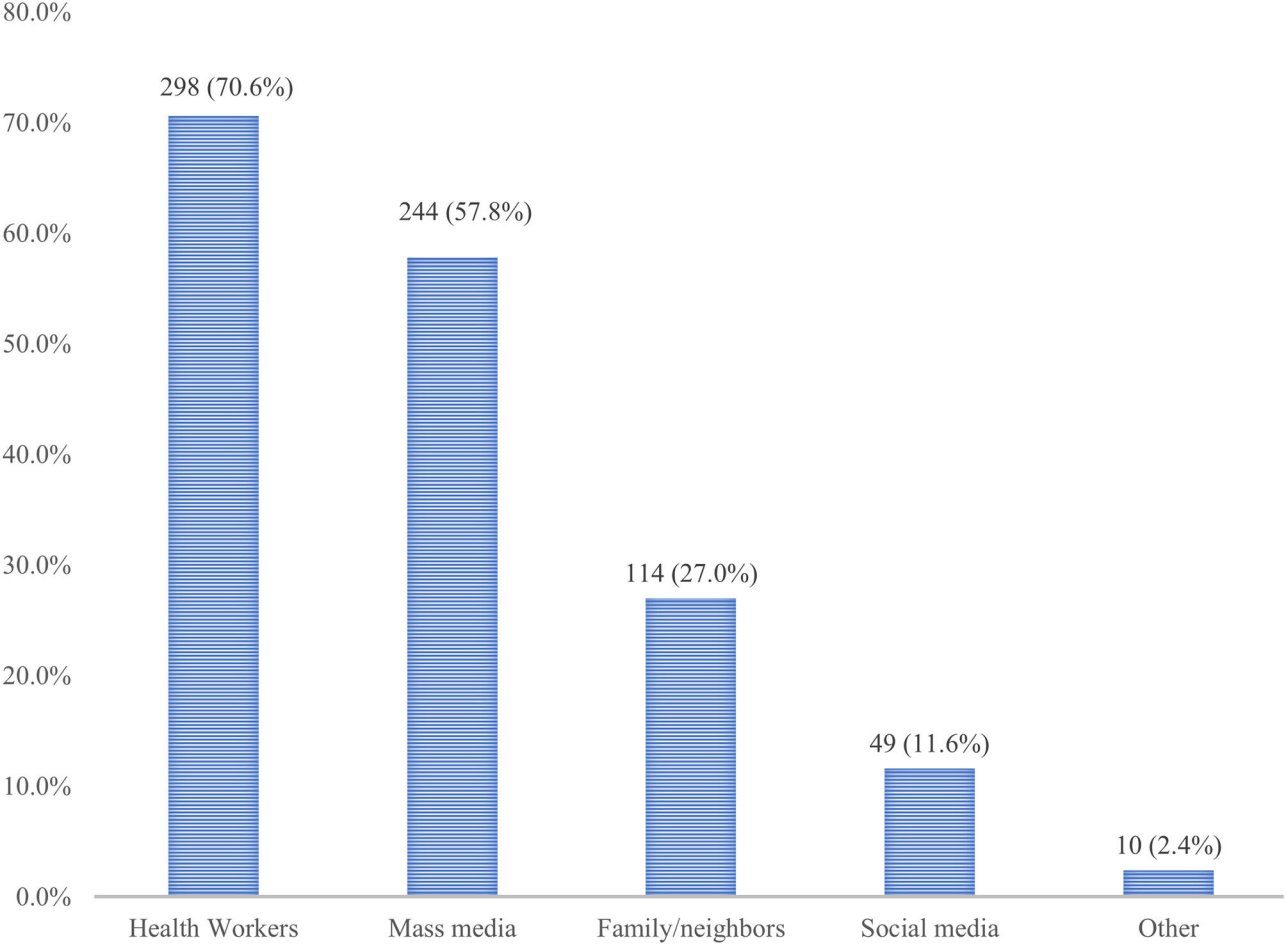
Source of information regarding breastfeeding among pregnant women attending MCH clinic of DTCSH in Debre Tabor, Northwest Ethiopia, 2022.

The BFKQ-A scores of the study participant ranged between 4 and 18, with a mean ±SD score of 11.0 ± 4.8. Two hundred forty-four (57.8%) of the study participants were evaluated to have adequate knowledge, the remaining 178 (42.2%), however, were below the median score and considered to have inadequate knowledge (Figure 1). Generally, the knowledge scale pertained to the domains of the benefits of breastfeeding to the baby, and effective feeding was the most high-scoring item (highest mean ± SD score). Whereas, the knowledge domains including the benefits of breastfeeding to the mother, problems with breastfeeding, colostrum, breast milk expression, breast engorgement, and the practical aspect of breastfeeding were shown to be low-scoring items (lowest mean **±** SD score). For instance, the majority of the study participant was not aware that breastfeeding reduces the risk of bleeding after childbirth and achieves pre-pregnancy weight faster with breastfeeding, which accounted for 71.3% and 75.2%, respectively. Similarly, about 79.3% of participants generally have poor knowledge regarding breast milk expression. A sizable proportion (61.7%) of women also did not know about the need for water after each breastfeeding. Another knowledge gap among participants was indicated by the belief that colostrum is difficult to digest by the baby and needs to be discarded, which took 45.5%. The main source of information for respondents regarding breastfeeding was health workers followed by mass media and family or neighbors, accounting for 298 (70.6%), 244 (57.8%), and 114 (27.0%), respectively (Figure 2).

### Attitude Toward Breastfeeding

The overall IIFAS-A score in our study ranged from 31 to 82, with a mean ± SD value of 54.7 ± 3.12. The overall attitude score indicated that only 198 (46.9%) of respondents had a positive attitude toward breastfeeding, while more than half 224 (53.1%) of them had a negative attitude toward breastfeeding (Figure 1). There were some IIFAS-A items with the highest mean ± SD, involving perceived breastfeeding as the ideal food for babies (90.1%), improving the health of the baby than formula-fed babies (87.1%), an important bonding factor between the mother and baby (86.4%), and one of the great joys of motherhood (83.1%). There were, however, some misconceptions explained with the lowest means ± SD and perceived as barriers to continuing breastfeeding. For example, nearly half (51.3%) of them agreed that a mother who occasionally consumes alcohol should not breastfeed her baby. Almost two-thirds (67.9%) of participants also disagreed that formula feeding is a better choice for women who plan to work.

### Intention to Practice Breastfeeding

The breastfeeding intention scores of the participants ranged between 3 and 8, with the mean ± SD of the intention scale being 5.4 ± 2.41. Overall, nearly two-thirds (65.4%) of study participants had a good intention to breastfeed, while the remaining (34.6%) were considered to have a poor intention (Figure 1). The study also showed that almost all (99.1%) participants intended to breastfeed their future child. But only a bit more than one-third (37.7%) of them had the intention to initiate breastfeeding within an hour of birth, while 41.0% will introduce breast milk after 1 h of birth and 21.3% were yet to decide. About 77.3% of participants intended to breastfeed exclusively, whereas 13.5% were still uncertain (Table 3).

**Table 3:** Breastfeeding intention among pregnant women attending the MCH clinic of DTCSH in Debre Tabor, Northwest Ethiopia, 2022.

**Variable**	**Category**	**Frequency (*n =* 422)**	**Percent (%)**
Plan to breastfeed your future child	Yes	418	99.1
	No	2	0.5
	Undecided	2	0.5
Intended time to initiate breastfeeding to your child	Within 1 h of birth	159	37.7
	After 1 h of birth	173	41.0
	Undecided	90	21.3
Intention to breastfeed your baby exclusively	Yes	326	77.3
	No	39	9.2
	Undecided	57	13.5
How will you breastfeed your baby	As mother's will	43	10.2
	As baby's demand	330	78.2
	Undecided	49	11.6
Intended time to start complementary feeding	<6 months	61	14.5
	After 6 months	317	75.1
	Undecided	44	10.4
Intentions to breastfeed for 10–20 min per feeding	Yes	225	53.3
	No	81	19.2
	Undecided	116	27.5
Intention to continue with breastfeeding up to 2 years	Yes	346	82.0
	No	60	14.2
	Undecided	16	3.8
Plan to practice bottle feeding	Yes	261	61.9
	No	74	17.5
	Undecided	87	20.6

### Factors Associated With Breastfeeding Knowledge, Attitude, and Intention

Maternal age, marital status, education, occupation, residence, gestational age, planned current pregnancy, medical illness during the current pregnancy, place of delivery, mode of delivery, parity, and breastfeeding history were entered into the logistic regression model and their associations with breastfeeding knowledge, attitude, and intention were first assessed using simple logistic regression analysis. Those variables with *p*-values ≤ 0.2 in the simple logistic regression were entered into multiple logistic regression models to control the possible confounders and to assess the factors associated with breastfeeding knowledge, attitude, and intention by expressing the results as AOR with 95% CI and *p*-values (Table 4). Accordingly, educational status, parity, number of ANC visits, and prior breastfeeding experience were significantly associated with the breastfeeding knowledge of participants. Participants who attended secondary education or college/university were two times (AOR = 2.0; 95% CI:1.31,3.19; *p* <0.05) and three times (AOR = 3.13; 95% CI: 1.63, 7.41; *p* <0.01), respectively, more likely to have adequate breastfeeding knowledge than those who did not attend any formal education. Multiparous women had two times (AOR = 2.11; 95% CI: 1.33, 3.43; *p* <0.05) more likelihood of having adequate knowledge compared to nulliparous mothers. Women with four or higher ANC visits had 1.45 times (AOR:1.45; 95% CI:1.21,4.31; *p* <0.05) more likely to have adequate knowledge than their counterparts. The odds of mothers who had prior breastfeeding experience having adequate knowledge was 3.53 times (AOR: 3.53; 95% CI: 2.22,5.65; *p* <0.01) more than those who never had experience.

**Table 4:** Factors associated with breastfeeding knowledge, attitude, and intention among pregnant women attending the MCH clinic of DTCSH in Debre Tabor, Northwest Ethiopia, 2022.

**Variables**		**Knowledge^a^**	**Attitude^a^**	**Intention^a^**
		**AOR (95% CI)**	**AOR (95% CI)**	**AOR (95% CI)**
Age (in years)	<25	1	1	1
	25–34	0.68 (0.36,1.27)	1.48 (0.79, 2.79)	0.73 (0.44, 1.42)
	35–44	0.69 (0.22, 2.25)	0.28 (0.03,3.01)	0.69 (0.53, 1.19)
	≥45	0.77 (0.29,1.98)	2.35 (0.66, 8.30)	1.44 (1.12, 5.59)^*^
Marital status	Married	1.1 (0.91, 3.4)	0.81 (0.36, 2.29)	0.97 (0.65, 1.66)
	Unmarried^b^	1	1	1
Educational status	No formal education	1	1	1
	Primary education	0.64 (0.11,3.89)	1.55 (0.96, 6.42)	1.32 (0.82,2.14)
	Secondary education	2.0 (1.31, 3.19)^*^	1.68 (0.82,3.46)	1.83 (0.39, 2.94)
	College/University	3.13 (1.63, 7.41)^**^	2.71 (2.33, 5.13)^*^	1.59 (0.84, 5.38)
Occupation	Housewife	1	1	1
	Other^c^	0.61 (0.22, 1.88)	1.78 (1.04,3.05)	1.87 (0.18, 4.09)
Residence	Urban	1	1	1
	Rural	1.68 (0.92, 3.08)	0.96 (0.58,1.59)	1.06 (0.34,3.71)
Gravidity	Primigravida	1	1	1
	Multigravida	1.84 (0.61, 4.22)	2.10 (0.89, 1.62)	2.07 (0.81, 4.62)
The current pregnancy is planned	Yes	1	1	1
	No	1.48 (0.82, 2.61)	0.87 (0.44, 1.58)	1.66 (0.74,3.71)
Parity	Null parous	1	1	1
	Primiparous	1.90 (0.27, 3.14)	2.11 (0.91, 4.83)	1.91 (0.79, 2.19)
	Multiparous	2.11 (1.33, 3.43)^*^	1.56 (1.32, 8.25)^*^	3.41 (0.87, 4.44)
Gestational age of current pregnancy	1st trimester	1	1	1
	2nd trimester	2.10 (0 0.75, 5.18)	3.63 (0.17,5.11)	2.51 (0.94, 6.19)
	3rd trimester	0.72 (0.61, 0.99)	1.29 (0.44,4.01)	1.16 (0.59,3.81)
Medical illness in current pregnancy	Yes	1	1	1
	No	1.22 (0.81,2.47)	0.66 (0.23,1.69)	0.78 (0.70,1.99)
Number of ANC visit	<4	1	1	1
	≥4	1.45 (1.21, 4.31)^*^	1.67 (0.60, 4.66)	1.7 (0.33, 3.7)
Place of delivery of their last child	Home	1	1	1
	Health institution	1.49 (0.82, 2.55)	1.62 (0.87, 2.97)	0.96 (0.44, 2.14)
Mode of delivery of their last child	Vaginal delivery	1	1	1
	Cesarean delivery	0.76 (0.65, 1.55)	1.21 (0.31, 4.71)	0.77 (0.45, 1.73)
Prior breastfeeding experience	No	1	1	1
	Yes	3.53 (2.22,5.65)^**^	2.08 (0.88, 3.47)	1.04 (0.84, 2.27)
Knowledge	Adequate	–	2.02 (1.88,7.14)^**^	5.21 (1.51,8.04)^**^
	Inadequate	–	1	1
Attitude	Positive	–	–	2.41 (1.50, 4.27)^*^
	Negative	–	–	1

a *AOR was interpreted by comparing adequate knowledge to inadequate knowledge, positive attitude to negative attitude, and good intention to poor intention*;

b *single, divorced, and widowed*;

c *government employee, private employee, merchant, daily laborer, and student*;

* *p-value < 0.05*;

** *p-value ≤ 0.01; 1, reference category; AOR, adjusted odds ratio; CI, confidence interval*.

The study also showed that maternal educational status, parity, and knowledge were significant predictors of breastfeeding attitudes. Women who attended college or university education were 2.71 times (AOR = 2.71; 95% CI: 2.33, 5.13; *p* < 0.05) more likely to have a positive attitude toward breastfeeding than those with no formal education. Compared to nulliparous women, multiparous women had 1.56 times (AOR = 1.56; 95% CI:1.32, 8.25; *p* < 0.05) greater likelihood of having a positive attitude. Likewise, the odds of having a positive attitude toward breastfeeding were about two times (AOR = 2.02; 95% CI:1.88,7.14; *p* < 0.01) higher in women with adequate knowledge than their counterparts. In addition, maternal age, breastfeeding knowledge, and attitude were observed to be statistically significant predictors of the intention to practice breastfeeding. Expectant women aged 45 years or above had 1.44 times (AOR = 1.44; 95% CI: 1.12, 5.59; *p* < 0.05) higher odds than those with age below 25 years. The odds of having good breastfeeding intention among women who had adequate knowledge were 5.21 times (AOR: 5.21, 95% CI: 1.51,8.04; *p* < 0.01) higher than those with inadequate knowledge. Those who had positive breastfeeding attitudes were a 2.41-fold (AOR = 2.41;95% CI: 1.50, 4.27; *p* < 0.05) greater likelihood to have good breastfeeding intention than those with a negative attitude.

## Discussion

Women begin motherhood with infant feeding knowledge and attitudes that they have adopted during their lives and make breastfeeding decisions before the birth of the child, prenatally if not pre-conceptually. Therefore, this study was aimed at evaluating the levels and determinants of breastfeeding knowledge, attitudes, and intentions among pregnant women. Our study revealed that about 57.8% of pregnant women had adequate knowledge regarding breastfeeding. This is nearly equivalent to the study done in Calabar, Nigeria (56.8%) (41), Gambia (60.2%) (32), and Moshi, Tanzania (61.2%) (42). But the result is larger than that of a similar study done in the Mana district of South West Ethiopia (52.4%) (36). These contrasting findings in the same country could be due to the difference in study settings in which the current study was conducted in those women who came to the hospital for ANC follow-up, who are expected to have good health-seeking behavior and may have a good opportunity to discuss with health professionals about breastfeeding and are more likely to have adequate knowledge. Besides, unlike our study, which was done in an urban area, the latter study was conducted in a rural community. Our result is also higher than the findings from Ibadan in Nigeria (43.1%) (28), Egypt (33.3%) (43), Lebanon and Qatar (25%) (33), and Northern Portugal (40%) (37), but it is lower than the figures from Nigeria (71.3%) (44), Saudi Arabia (86%) (45), and Jordan (78%) (38). This may be attributed to the difference in media exposure, sociodemographic variables (such as education), and knowledge assessment scale and criteria.

In the current study, educational status, parity, number of ANC visits, and prior breastfeeding experience were independently associated with the breastfeeding knowledge of pregnant women. Adequate breastfeeding knowledge was more likely found in women with higher education compared to those who did not attend any formal education. Although the study by Omoronyia et al. (41) observed no significant correlation, several prior studies have shown educational status as a significant predictor of breastfeeding knowledge (37, 46). This may be because women who had attained higher education can easily be well informed about breastfeeding and hence acquire knowledge (20). Thus, pregnant women with no formal education should be given special attention and receive extensive breastfeeding advice during their prenatal visit, as they are less likely to acquire breastfeeding knowledge.

This study further indicated that the parity of women was significantly associated with the level of breastfeeding knowledge, with multiparous women having a more likelihood of having adequate knowledge than their counterparts. In contrast, there was no significant association between the level of knowledge and the parity of women (41). Another study undertaken in Nigeria, on the other hand, reported similar results to our findings (40). Consistently, prior breastfeeding experience was reported to be a significant predictor of adequate knowledge of respondents. This is likely due to the more experiences of motherhood, the more likelihood of having adequate breastfeeding knowledge. This study also reported that mothers with four or higher ANC visits had significantly more odds of having adequate knowledge and this corroborated with a prior study conducted in Southwest Ethiopia (36). The likely explanation is that ANC visits create the best opportunities for pregnant women to get appropriate key breastfeeding messages from the health personnel and thus, women with a greater number of ANC follow-up may have more likelihood to get breastfeeding education. However, this finding is inconsistent with the study done in Nigeria that shows no significant association between ANC visits and breastfeeding knowledge (40).

The overall positive attitude of participants toward breastfeeding in this study was 46.9%. This value is higher than the study finding in Nigeria (7.4%) (41), Southwest Ethiopia (36.9%) (36), and Lebanon and Qatar (9.2%) (33). However, it is lower than the result from Saudi Arabia (62.2%) (47), South West Nigeria (53.8%) (28), and Jordan (72%) (38). The use of different tools and criteria to categorize attitude levels, as well as differences in sociodemographic factors, could explain this discrepancy. Accumulated evidence indicates a positive relationship between attitude and optimum breastfeeding practices (33, 45, 48). The attitude of mothers toward breastfeeding predicts appropriate breastfeeding practices of the expecting mothers, and the attitudes could in turn are affected by various factors. In this study, educational status, parity, and breastfeeding knowledge were found to be significant predictors of their attitude.

Educational status is an independent predictor of a positive attitude toward breastfeeding, implying that women with higher education had more odds of having a positive attitude than those with no education. This is in line with previous studies conducted in Lebanon (49), Ireland (50), China (51), Singapore (52), and Spain (53). A prior study in Ethiopia also indicates that women with lower educational status had a lower attitude score, though not significant (36). One possible explanation is that women with higher educational levels are more likely to adopt an infant feeding attitude *via* proactive information-seeking behavior that changes their attitudes (17, 54). This study further found that multiparous women had a more positive breastfeeding attitude than nulliparous women, which keeps up with another study done in China (51). This is more likely as a result of multiparous women with better information or prior breastfeeding experience would positively reinforce their attitudes. This is opposing another study done in Southwest Ethiopia, reporting that multiparous women have less positive attitudes than their counterparts (36). This discrepancy could be due to the difference in the study settings. Our study was conducted in the urban area as opposed to the rural area in the earlier study, and so mothers are expected to be less busy with work compared to rural women.

Women with adequate breastfeeding knowledge were also a significant determinant of positive attitude toward breastfeeding. This finding is consistent with a survey done in Saudi Arabia (55), the US (56), Ethiopia (36), and Nigeria (28). This corroborates the theory of the knowledge-attitude-behavior paradigm, which assumes that knowledge of individuals' health is a key factor in engaging in a particular health-related behavior (57). In particular, pregnant women with adequate knowledge can better understand the potential benefits of breastfeeding, resulting in a favorable breastfeeding attitude. Conversely, women with inadequate levels of knowledge may not comprehend the benefits of breastfeeding; instead, they may overconfidently stick to their socio-culturally based misconception (58, 59). But a study from Calabar, Nigeria, documented no significant relationship between infant feeding knowledge and attitude (41).

The study also reported that the intention to practice breastfeeding among pregnant women was 65.4%, suggesting that a greater proportion of women would have a less likelihood to appropriately breastfeed their expecting babies. This figure is lower than the study done in the US (73%) (34), Jordan (75%) (60), and even more significantly lower than Australia (95%) (19), China (99.1, 73.2, and 92.17%) (46, 61, 62), Saudi Arabia (80.8%) (63), and Brazil (96.5% and 98.5%) (64–66). But our finding is higher than the findings from the US (53%) (35), Nigeria (35.7%) (28), and Gambia (38.6%) (32). Differences in intention measuring scales and categories, variation in sociodemographic variables, discrepancies in breastfeeding promotion, and differences in study participants may explain these disparities.

Breastfeeding intention is a well-known determinant of breastfeeding practice that can be influenced by a variety of interwoven factors (21–24). In this study, the main identified factors that influenced breastfeeding intention were age, breastfeeding knowledge, and attitude. It has been found that older women had higher odds of intending to breastfeed than younger ones. This is corroborated by other studies that show that advanced maternal age is a significant predictor of good breastfeeding intention (60, 63, 64). This might be explained by the fact that breastfeeding is, in many aspects, learned behavior and so old mothers are likely linked to prior breastfeeding experience that influences their intention to breastfeed. In contrast, other reports demonstrate that older women are less likely to breastfeed (32, 40, 67, 68). Another study revealed no association between maternal age and breastfeeding intention (35).

The current study also revealed that breastfeeding knowledge is significantly associated with the intention to breastfeed. Women with adequate knowledge are more likely than their counterparts to have a good breastfeeding intention, confirming that prenatal breastfeeding knowledge has a significant influence on breastfeeding decisions. This agrees with other studies, showing a link between prenatal breastfeeding knowledge and intention (19, 22, 28, 60, 69). Attitude is a measurable factor that has been shown to influence behavior through intention (70). According to the planned behavior theory, along with subjective norms and perceived behavioral control, attitude is suggested to be a strong predictor of maternal intention to breastfeed. In turn, the intention is thought to be a key determinant factor of immediate behavior. This link between attitude and intention has been established by numerous prior studies for different health-related behaviors, such as infant feeding practice (19, 20, 22, 26, 32, 38, 71, 72). Similarly, we found a significant association between prenatal breastfeeding attitude and intention, with those who had positive attitudes having a good intention to breastfeed than those with negative attitudes.

## Limitation of the Study

This study has certain drawbacks, despite its inherent strengths. One is that the study employed a cross-sectional study design that inherently limits the temporal relationship between cause and effect. The actual breastfeeding practice and its determinants were not assessed since the study was conducted among pregnant women and was not prospective. Besides, the study findings may be limited in the representativeness of the general population due to the small sample size, use of convenience sampling method, and a hospital-based study.

## Conclusion

In general, the level of adequate knowledge and positive attitudes toward breastfeeding were suboptimal. The overall breastfeeding intention of pregnant women was also unsatisfactory, suggesting that women would have a lower likelihood of breastfeeding their expecting babies appropriately. Educational status, parity, number of ANC visits, and prior breastfeeding experience were identified as significant determinants of breastfeeding knowledge. Whereas, educational status, parity, and knowledge were found to be significant predictors of breastfeeding attitude. Age, breastfeeding knowledge, and attitude, on the other hand, were observed to be significantly associated with the intent to practice appropriate breastfeeding.

Since breastfeeding intention is an immediate predictor of future behavior, more emphasis needs to be directed toward breastfeeding promotion during the prenatal period. Although the road between intention and behavioral outcomes is hard, breastfeeding knowledge and attitudes can be changed and improved through prenatal education. Thus, the authors strongly believe in the need for continuous education for pregnant women by healthcare personnel during their ANC visits to encourage breastfeeding practice. This highlights the need for policymakers and managers to devise culture-specific interventions and policies aimed at improving women's breastfeeding knowledge and attitudes, and hence their intention to breastfeed their future babies. We also suggest that large-scale, community-based follow-up studies should be done to assess the impact of these factors on actual breastfeeding practice after a child's birth.

## Data Availability Statement

The raw data supporting the conclusions of this article will be made available by the authors, without undue reservation.

## Ethics Statement

The studies involving human participants were reviewed and approved by the Ethical Review Committe of Debre Tabor University. The patients/participants provided their written informed consent to participate in this study.

## Author Contributions

EC contributed to study conception and design and manuscript drafting. GAy, GAs, and TA were involved in manuscript preparation, tables and figures, and critical revision.TM, ZT, AB, and AT contributed to the literature search and manuscript writing. EC, GAy, GAs, TA, TM, ZT, AB, and AT involved in data analysis. TM, ZT, AB, and AT contributed to the interpretation. All authors were involved in reviewing the final draft of the manuscript, revision of the manuscript, and approved the submitted version.

## Conflict of Interest

The authors declare that the research was conducted in the absence of any commercial or financial relationships that could be construed as a potential conflict of interest.

## Publisher's Note

All claims expressed in this article are solely those of the authors and do not necessarily represent those of their affiliated organizations, or those of the publisher, the editors and the reviewers. Any product that may be evaluated in this article, or claim that may be made by its manufacturer, is not guaranteed or endorsed by the publisher.

## Abbreviations

ANC: Ante Natal Care
AOR: Adjusted Odds Ratio
BFKQ-A: Breastfeeding Knowledge Questionnaire-Amharic version
CI: Confidence Interval
DTCSH: Debre Tabor Comprehensive Specialized Hospital
EBF: Exclusive Breastfeeding
IIFAS-A: Iowa Infant Feeding Attitude Scale Amharic version
MCH: Maternal and Child Health
SD: Standard Deviation.
